# Viruses of parasites: A roadmap toward diagnostic and therapeutic development

**DOI:** 10.1371/journal.pntd.0012982

**Published:** 2025-04-10

**Authors:** Sarah Temmam, Nolwenn M. Dheilly

**Affiliations:** 1 Pathogen Discovery Laboratory, Institut Pasteur, Paris, France; 2 Institut Pasteur, Université de Paris Cité, The WOAH (OIE) Collaborating Center for the detection and identification in humans of emerging animal pathogens, Paris, France; Oregon State University College of Veterinary Medicine, United States of America

## Abstract

With few preventive and therapeutic solutions available, parasites remain associated with devastating health, social and economic consequences, especially in impoverished communities in tropical areas. The discovery that parasites host viruses, and that these parasite viruses can contribute to diseases, has triggered a paradigm shift in thought and action, whereby parasite viruses are being assessed as targets for diagnostic, therapeutic and preventive interventions. This review lays out critical steps needed to discover and characterize viruses of parasites, highlighting challenges and identifying opportunities through examples of virus discoveries that fill the gap in our incomplete understanding of parasite pathogenicity.

## Introduction

Parasites, such as helminths and protozoans but also fungi and arthropods are responsible for a diverse range of diseases that continue to burden human and animal health worldwide, with major direct and indirect economic consequences. We are currently witnessing a shift in the parasitology community that is moving from studying the virulence and resistance factors that mediate parasite–host interaction, toward the study of the role of microbes associated with the host and with the parasite in the outcome of infection. The driving hypothesis proposed by the Parasite Microbiome Project consortium is that phenotypes, symptoms, and evolutionary dynamics that could not be explained through traditional approaches may result from microbes associated with the host, or with the parasite [1–9].

**Fig 1:**
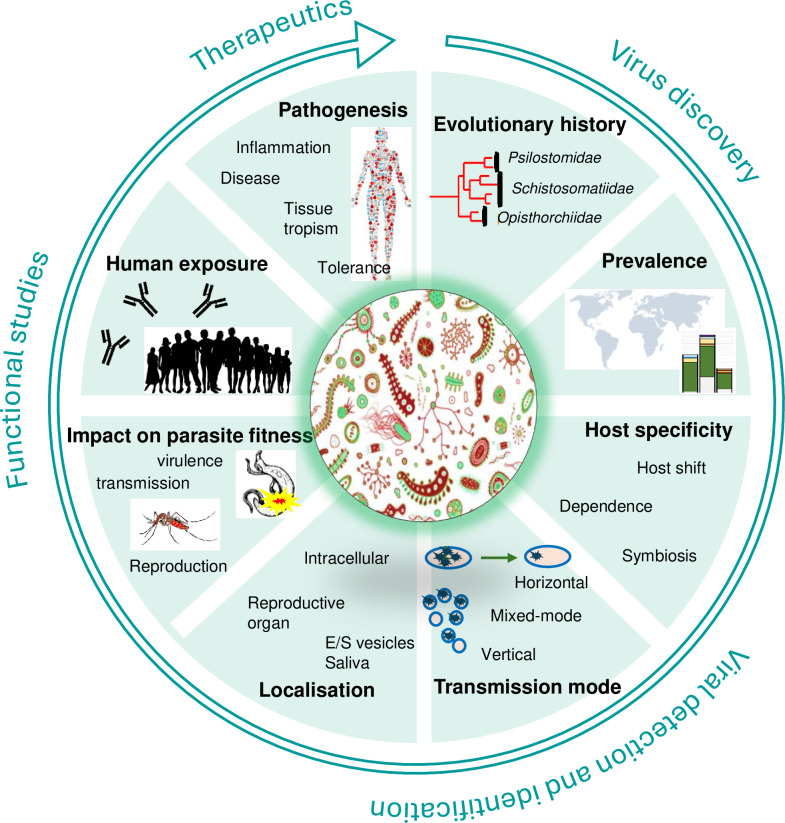
Roadmap for a comprehensive characterization of a novel parasite virus from virus discovery to the development of detection and identification methods, functional studies, and identification of therapeutic applications. Studies needed encompass all fields of virology such as the study of the biology of viruses and viral diseases, including the evolution, distribution, ecology, physiology, tropism, molecular biology, epidemiological, and clinical aspects of viruses in order to provide a complete picture of the virus–parasite (co-)evolutionary history and of the virus role in parasite–host interaction.

The metagenomics sequencing revolution has revealed that viruses are ubiquitous. They infect almost every eukaryotic and prokaryotic species and are the most abundant biological entities on Earth. Parasites are no exception. Arthropods are well-known vectors of viruses of health significance (known as arboviruses), but nematodes, flatworms, fungi, and protozoans also host a plethora of viruses, hereafter called parasite viruses [10–18]. Remarkably, some parasite viruses are transmitted from the parasite to the vertebrate host, stimulate the host immune response, and contribute to modulating parasite pathogenicity [11,13,19–21]. These noteworthy discoveries are leading toward the rise of a novel field of research focusing on targeting parasite viruses for parasite detection [22–24] and on characterizing their role in modulating parasite pathogenicity [10,11,19,25–28]. Despite the relative youth of the field, it is becoming evident that parasito-virology is offering new perspectives for the development of diagnostic, preventive, and therapeutic strategies with the potential to revolutionize the fight against parasites, many of which being responsible for major neglected tropical diseases [29]. The discovery of the ubiquity of parasite viruses coincides with a time when the field of virology is benefiting from remarkable technological advancements that facilitate the study of non-cultivable viruses. In this viewpoint, we consider the challenges associated with the study of parasite viruses and provide a roadmap of key methodologies and detection tools to develop, and biological data to collect to assess how each parasite virus discovery can be best exploited in the fight against parasitic diseases (Fig 1).

### Virus discovery

As obligate intracellular parasites, viruses require cells in which to replicate. The cells must express appropriate receptors and other proteins required by the virus. Cultured cells are often used to study basic steps in viral replication [30]. Viruses can be purified away from cellular proteins and organelles using centrifugation techniques [31]. Historically, the process of obtaining a pure virus culture was a necessary first step toward virus characterization [32]. In permissive cells, virally induced characteristic changes to cell morphology (known as cytopathic effects) are observable under light microscopy. Novel virions are released in the supernatant, producing a virus culture that can be purified.

For most parasites, the presence of associated viruses has never been investigated. The isolation of parasite-borne viruses—namely viruses of parasites that can infect parasitized hosts—may be attempted on vertebrate cell lines. Parasite-specific viruses isolation may prove more difficult given that parasite cell lines are not available, but *in vitro* culture of parasite isolates infected by known viruses may be considered instead.

Fortunately, parasito-virologists can rely on next generation sequencing (NGS), a technology that has revolutionized virology by facilitating virus discovery [13,14,33]. Indeed, conversely to bacteria, viral genomes are highly variable in size (from less than 5 kb to 1.2 Mb) and nucleic composition (single- or double-stranded monopartite or segmented DNA or RNA molecules), and no conserved genes exist across viral taxa, preventing the development of generic methods of identification. Therefore, most recent virus discoveries rely on sequence-independent (or random) methods of amplification followed by NGS, known as metagenomics (sequencing of the total DNA content of a sample) or metatranscriptomics (sequencing of the total RNA content of a sample) [34]. Yet, the detection of highly divergent viral sequences, which relies on sequence comparison to public nucleotide and protein databases, is limited to viruses with a minimum of 30% of amino-acid identity to known viral sequences. This limitation impacts parasite virus discovery because our current knowledge of virus taxonomy is strongly biased toward viruses that infect humans, arthropods, bacteria, and land plants and because parasite viruses often belong to novel distant taxa [13,35–37]. That being said, recent developments in using deep learning algorithm for the purpose of virus discovery is showing great promise [38]. Finally, upon virus genome discovery, assembly, and annotation, the parasitology community must commit to submitting newly discovered viruses to be approved and ratified by the International Committee on Taxonomy of viruses (https://ictv.global/taxonomy/) to allow quality control, taxonomic classification of the newly discovered viruses, and inclusion in reference databases [39].

Even though virome studies do not suffer from the same level of risk of contamination as microbiome studies [40], confirmation of virus–host association is encouraged. The most straightforward approach is to collect phylogenetic evidence that the newly discovered virus is phylogenetically related to viruses discovered in closely related parasite species. Indeed, evidence shows that parasites host novel taxa of viruses, and that parasite viruses often co-diversify with their parasitic hosts (Fig 1, evolutionary history) [13]. Because the RNAi response is a conserved and critical pathway to control virus replication in invertebrates, the production of viral short interfering RNAs (typically 22–23 nt long small RNAs in nematodes) can also be used to confirm virus association with the parasitic host [41]. Complementary approaches rely on the development of virus detection methods to confirm virus presence in different individuals, life stages or populations, and to visualize viral RNA or viral proteins within the parasite individual.

### Virus detection and identification

Upon virus discovery, the development of a reliable PCR-based molecular diagnostic tool is a necessary first step to conduct epidemiological studies of the distribution of parasite viruses within the parasite geographic distribution. Polymerase Chain Reaction (PCR) [42] method is a simple, rapid, and inexpensive method to detect the viral genome either directly for DNA viruses or following a retro-transcription for RNA viruses. While the basic principle of PCR is simple, the development of PCR diagnostic tools with specific properties can be challenging and require multiple steps of validation to ensure high sensitivity and the predetermined necessary level of specificity. Indeed, it is important to consider the sometime high genetic diversity of RNA viruses, and the potential for cross-reactivity among close relatives. Depending on objectives, it may be useful to develop a single PCR diagnostic tool with broad diagnostic sensitivity to closely related viruses (often known as pan-generic PCR), or to develop a panel of highly specific PCR diagnostic tools that each detect a single virus. PCRs have already demonstrated their use to determine parasite virus prevalence, test parasite virus vertical transmission, and assess transmission to the parasitized host (Fig 1, prevalence, host specificity, transmission mode) [20–23,41].

Antigenic tests that detect proteins expressed on the surface of viruses can be useful tools to help manage epidemics. Some parasite viruses appear to reach 100% prevalence in their parasitic host and can therefore be targeted as markers of a parasite infection in the parasitized vertebrate host. For example, the apicomplexan *Cryptosporidium parvum*, an intracellular parasite responsible for moderate-to-severe diarrhea, hosts a dsRNA Cryspovirus of the family *Partitiviridae* [43–46] with such a high prevalence in the parasite population (basically 100%) that molecular diagnostic methods and antigenic methods have been developed to detect the Cryspovirus genome and protein, respectively [22–24]. RT-PCR methods could detect less than five *C. parvum* oocysts in calf feces when nested PCR were needed to detect the *C. parvum* DNA [22]. Colloidal gold strips allowed the detection of about 16 oocysts per gram of feces sample, which represents a major improvement compared to the microscopic detection method that has a sensitivity of 1.4 × 10^6^ oocysts per gram [23].

Microscopy techniques have been instrumental in our incremental comprehension of virus structure, pathogenesis, and interaction with their hosts [31]. Static images of viruses can be obtained through conventional scanning electron microscopy and transmission electron microscopy. Fluorescence microscopy has allowed virologists to obtain a more dynamic image of virus interaction with the host cell. Immuno-histochemistry, that relies on antibodies raised against specific proteins of the virus, is used extensively to localize viruses within cells, but the lack of sensitivity and cost of custom antibody production remains a limitation. An alternative has been the insertion of Green Fluorescent Protein (GFP) or enhanced GFP into viral genomes to study intracellular trafficking in real time [47]. Finally, *in situ* hybridization remains extensively used to localize specific RNA or DNA viral nucleic acids within cells. Most recently, RNA in situ hybridization (ISH) technology has allowed highly sensitive, specific, and rapid detection of viral genome within cells. Notably, virus localization remains a gold standard practice to demonstrate virus–host association and can provide information on the virus transmission mode (Fig 1, localization) [12,15]. For example, the Brugia malayi RNA virus 1 was localized in the reproductive tissues of the Filarial nematode *Brugia malayi* suggestive of vertical transmission, but also within epicuticular inflations that could explain transmission to parasitized jirds [41].

Seroconversion is the production of specific antibodies in the vertebrate blood serum in response to an infection or vaccination. Serological methods that aim at quantifying these antibodies are extensively used in virology to assess human exposure (Fig 1, human exposure). Various methods have been developed including hemagglutination inhibition, Enzyme-linked immunosorbent assay (ELISA) or immunofluorescence assay [48]. Seroneutralization that requires the isolation of viruses (and often requires manipulation in BSL3 labs) remains the gold standard. It consists of incubating permissive cell lines and target virus in the presence of different dilutions of serum and measuring disappearance of cytopathic effects (in the presence of neutralizing antibodies) or virus replication. If neutralizing antibodies are present in the serum, they block the virus before it can infect the cells.

Pseudotyped viral particles are a good alternative for uncultivable viruses. Pseudotypes are chimeric viruses based on a backbone virus (like a lentivirus or a vesiculovirus) in which the structural protein has been replaced by the one from the target virus, and in which a reporter gene has been added. Pseudotypes cannot replicate in competent cells, allowing their use in BSL2 labs. The pseudoneutralization test resembles seroneutralization except that the presence of neutralizing antibodies is assessed by measuring the signal of the reporter carried by pseudotyped viruses [49]. Alternatives that can be implemented rapidly in a molecular lab are Luciferase ImmunoPrecipitation System (LIPS) and Luciferase-linked Immunosorbent assays (LuLISA) [50–53]. LIPS consists of expressing viral targets in fusion with a nanoluciferase reporter and use the fusion protein as antigens in an immunoprecipitation reaction. LuLISA consists in an indirect ELISA in which the primary antibody is detected by an antibody fragment from camel (i.e., llama or alpaca) known as VHH (Variable Heavy domain of Heavy chain) expressed in tandem with a shrimp luciferase [54,55]. In both cases, the activity of the luciferase is assessed by measuring the production of light and proportional to the amount of antigen-binding antibodies. Quek et al [41] recently provided the first evidence of human seropositivity against a parasite virus: parasitized and exposed individuals from Uganda, Togo, Nigeria, Cameroon, and Ecuador had high level of seropositivity against Onchocerca volvulus RNA virus 1, a novel rhabdovirus associated with the Filarial nematode.

### Functional studies of parasite-associated viruses

The next step following virus identification and characterization is to determine the extent to which viruses contribute to the outcome of a parasitic infection. The significance of the parasite virome spans not only parasite-borne viruses that are infecting the host, but also parasite viruses strictly associated with the parasite which might modulate parasite virulence, reproduction, and survival to ensure their own viral transmission. Within this nested context, fulfilling Koch’s and Rivers’ postulates to establish causation may prove difficult [56]. Carefully planned experimental designs are therefore necessary to determine which viruses are transmitted to parasitized host, modulate host immune response and susceptibility to infection, or alter the parasite fitness and virulence in its host (see figures in [8,9]).

As discussed above, the development and optimization of protocols for *in vitro* culture of parasite cells would facilitate controlled experiments to address hypotheses underlying parasite–virus interactions. *In vivo* culturing of parasite developmental stages [57–61] represents a readily available alternative that, combined with genome editing technologies [62–65], can be used to functionally assess virus–host proteins interactions. As a complementary tool to assess effects of individual viruses on both the host and parasite fitness (Fig 1, impact on parasite fitness, pathogenesis) through controlled experiments, virus-free or gnotobiotic (individuals exclusively colonized by known viruses) parasites might be collected in the field, or generated via antiviral treatments.

Should a parasite virus be one day isolated in cell culture, a large panel of vertebrate cells and organoids could be tested to assess tissue tropism and characterize the virus–host interaction at the molecular level. Regardless, and when feasible for the parasite under study, animal experiments can provide insight on a parasite virus ability to transmit to the parasitized host, tissue tropism, and role on modulating host immune response [11,13,19–21]. Experimental infection of Threespine sticklebacks with the cestode *Schistocephalus solidus* first demonstrated the transmission of a flatworm parasite-associated rhabdovirus to a vertebrate [21]. Soon after, the screening of parasite viruses in a diversity of flatworm species revealed that rhabdoviruses of flatworms are ancestral to rabies virus and other *Alpharhabdovirinae*, indicating that they emerged at least once in a parasitized vertebrate host [13]. Animal experiments were also instrumental in demonstrating the association between the Type I IFN pathway activated by Leishmania RNA virus 1 and the pathogenicity of *Leishmania guyanensis* and *Leishmania braziliensis* [11,19]. Similarly, using immunocompromised mice, Deng and colleagues [20] showed that Cryptosporidium parvum virus 1 delivery into parasitized epithelial cells activates type I IFN signaling, which in turn attenuates the IFN-γ-mediated protective response.

## Conclusion

To conclude, parasite virus discoveries and characterizations are contributing to a paradigm shift in parasitology with investigations reaching beyond the study of virulence and resistance factors that mediate parasite–host interaction toward the study of the role of associated microbes in the outcome of parasite infection [1–7]. Within this framework, parasite viruses are microbial targets whose role in the dynamic crosstalk between parasite and host can be readily investigated and exploited thanks to the broad range of virology tools and approaches available. Should a parasite-borne virus show an active role in parasite pathogenicity, its susceptibility to antivirals could also be assessed *in vitro*, providing opportunities for novel therapeutics, and novel vaccines could be developed to provide protection against diseases caused by the virus itself. We expect the discovery and characterization of virus role in parasite pathogenicity will allow major advance in diagnostic and therapeutic development and may provide opportunities for improved prevention against parasitic diseases.
